# Comparison of pregnancy outcomes among patients of different ages who underwent frozen-thawed high-quality single blastocyst transfer

**DOI:** 10.1186/s12884-024-06451-w

**Published:** 2024-04-15

**Authors:** Haoying Chen, Shenghao Wu, Weijue Su, Junzhao Zhao, Yanhong Wu

**Affiliations:** Department of Reproductive Center, Obstetrics and Gynecology, The Second Affiliated Hospital and Yuying Children’s Hospital of Wenzhou Medical University, Wenzhou, China

**Keywords:** Advanced maternal age (AMA), Assisted reproductive technology (ART), Frozen-thawed embryo transfer (FET), Pregnancy outcomes, Single blastocyst transfer (SBT)

## Abstract

**Objective:**

To investigate the feasibility of performing frozen-thawed high-quality single blastocyst transfer in women of different ages.

**Methods:**

A total of 1,279 women were divided into four groups: a 38-40-year-old group (*n* = 147), 35-37-year-old group (*n* = 164), 30-34-year-old group (*n* = 483), and < 30-year-old group (*n* = 485). Intergroup comparisons of baseline characteristics and pregnancy and neonatal outcomes were made.

**Results:**

The clinical pregnancy rate (47.6%), and live birth rate (34.0%) in the 38-40-year-old group were significantly lower than those in the 30-34-year-old group (64.4%, 50.9%, respectively; all *P* < 0.001) and < 30-year-old group (62.9%, 50.7%, respectively; all *P* < 0.001). However, the 35-37-year-old group did not differ from the other three groups in these two dimensions (all *P* > 0.05). Moreover, there were no differences in the rates of biochemical pregnancy, miscarriage, or obstetric or neonatal complications among the four groups (all *P* > 0.05). According to the multivariate logistic regression analysis, the 35-37-year-old group was not associated with non-live birth outcomes, adverse pregnancy outcomes, or obstetric or neonatal complications. However, being 38–40 years of age was a risk factor for non-live birth (OR = 2.121, 95% CI: 1.233–3.647) and adverse pregnancy outcomes (OR = 1.630, 95% CI: 1.010–2.633). Post hoc power analysis showed that the study was sufficiently powered to detect meaningful differences.

**Conclusion:**

Frozen-thawed high-quality single blastocyst transfer produces the same satisfactory pregnancy outcomes for women aged 35–37 years as younger patients. Future prospective randomized controlled studies with larger populations are needed to verify the feasibility and safety of this method.

**Supplementary Information:**

The online version contains supplementary material available at 10.1186/s12884-024-06451-w.

## Introduction

The Chinese Society of Reproductive Medicine (CSRM) proposed single embryo transfer (SET) in the Chinese Expert Consensus on Numbers of Embryos Transferred to reduce the risk of adverse maternal and infant outcomes in women undergoing assisted reproductive technology (ART) cycles for the first time in 2018. This transfer strategy is considered the best choice for reducing multiple pregnancy rates and improving perinatal outcomes [1]. Unfortunately, the consensus stated that SET is only appropriate in certain exceptional circumstances. Three years later, in the Chinese Practice Guideline on ART Strategies for Women with Advanced Age [2], the CSRM recommended that selective SET be used for women aged 35–37 years with a good prognosis (1A), and that double embryo transfer (DET) be considered for women with a poor prognosis or aged > 37 years (2B). Although the guidelines fill gaps in embryo transfer strategies for women of advanced age, unfortunately, there are barriers to implementing this strategy due to a lack of standardized clinical practice. With the implementation of the two-child policy in 2015 and the three-child policy in 2021, the demand for ART for women of advanced age in China has soared. How to help women of advanced age achieve pregnancy safely and efficiently is a serious challenge for reproductive doctors worldwide.

Compared with fresh cleavage-stage embryo transfer, fresh blastocyst transfer can improve pregnancy outcomes [3]. Due to improvements in laboratory quality control, good culture environments and freezing technology, single blastocyst transfer (SBT) is widely used in clinical practice. Our team previously conducted a study on the number and type of blastocysts transferred and found that high-quality SBT was the optimal frozen-thawed embryo transfer (FET) strategy for young women [4]. Although this strategy is still feasible for patients aged 35–40 years [5], there were some limitations in the previous study; for example, women of advanced age were not stratified, and it is impossible to determine whether the benefits of SBT are the same for women aged 35–37 years and older. To our knowledge, no studies have explored on age stratification in frozen-thawed high-quality SBT. Therefore, women of advanced age were stratified in this study according to guidelines and based on previous studies by this team with the aim of optimizing FET strategies for women of different ages and providing evidence for reliable FET strategies for women of advanced age.

## Materials & methods

### Research objects

A retrospective analysis of women who underwent FET at the Reproductive Center of the Second Affiliated Hospital of Wenzhou Medical University from January 2018 to December 2021 was performed. The inclusion criteria were as follows: (1) age ≤ 40 years; (2) an endometrial thickness ≥ 7 mm on the day of endometrial transformation; (3) no more than 2 transfer cycles; (4) the transfer of a single high-quality blastocyst on day 5; and (5) an endometrial preparation protocol involving hormone replacement therapy (HRT). The exclusion criteria were as follows: (1) abnormal ultrasound findings in the uterus, such as endometrial polyps, uterine fibroids, uterine adhesions, adenomyosis, or reproductive tract abnormalities; (2) malignancy or other systemic chronic diseases, including autoimmune or hematologic conditions; (3) a history of genetic disease in one of partners of the treated couple; (4) a history of recurrent miscarriage or recurrent implantation failure; and (5) preimplantation genetic testing of the blastocysts.

**Fig. 1:**
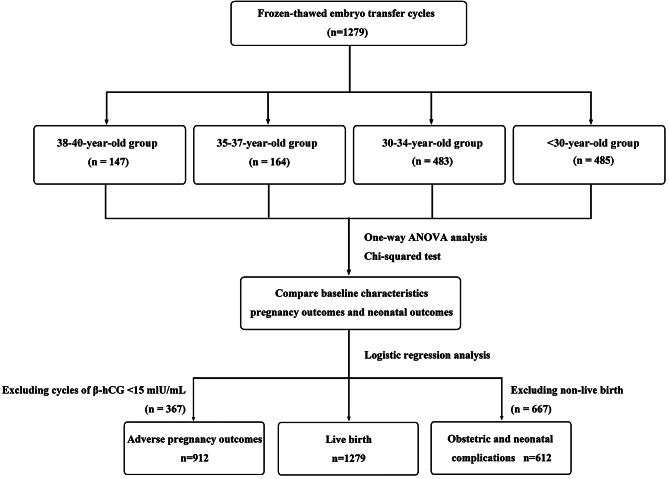
Flow chart. A total of 1,279 eligible patients who underwent frozen-thawed embryo transfer (FET) at the Reproductive Center of the Second Affiliated Hospital of Wenzhou Medical University from January 2018 to December 2021 were included. The infertile women were divided into four groups according to their age: a 38-40-year-old group (*n* = 147), 35-37-year-old group (*n* = 164), 30-34-year-old group (*n* = 483), and < 30-year-old group (*n* = 485). Statistical analysis was used to compare patient data (Fig. 1)

A total of 1,279 eligible patients were enrolled and divided into four groups according to the age of the infertile women: a 38-40-year-old group (*n* = 147), 35-37-year-old group (*n* = 164), 30-34-year-old group (*n* = 483), and < 30-year-old group (*n* = 485) (Fig. 1).

### Endometrial preparation protocol

**Fig. 2:**
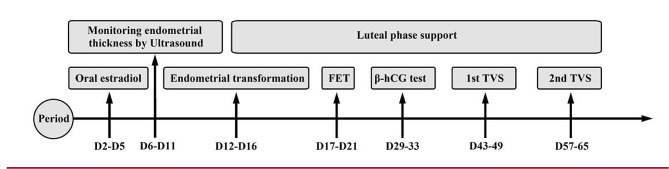
Endometrial preparation protocol and follow-up. Hormone replacement therapy patients were started on estradiol from day 2 to day 5 of the menstrual cycle. Endometrial thickness was monitored by ultrasound every 3–5 days, and the dose of estradiol was adjusted according to the endometrial thickness. When the endometrial thickness was greater than or equal to 7 mm and the progesterone concentration was less than 1.2 ng/mL, progesterone was given to initiate endometrial transformation. Single high-quality blastocyst transfer was performed on day 5 after endometrial transformation. Luteal phase support after transfer was consistent with endometrial transformation. Pregnancy was determined by β-human chorionic gonadotropin (β-hCG) testing on days 10–12 after FET. The first B-ultrasound was performed 14–16 days after β-hCG testing, and the second B-ultrasound was performed 14–16 days after the first B-ultrasound (Fig. 2)

The HRT patients took one estradiol tablet (Femoston; Abbott Biologicals B.V. Dose: 2 mg estradiol/tablet) orally twice daily from day 2 to day 5 of the menstrual cycle. Endometrial thickness was monitored by ultrasound every 3–5 days, and the estradiol tablet dose was adjusted according to the endometrial thickness. When the endometrial thickness was greater than or equal to 7 mm and the progesterone level was less than 1.2 ng/mL, 10-mg oral dydrogesterone tablets (Duphaston; Solvay Pharmaceuticals B.V. dose: 10 mg/tablet) and 200-mg progesterone soft capsules (Utrogestan; Capsugel, Besins Manufacturing Belgium, Bruxelles, Belgium; dose: 0.1 g/tablet) were administered orally or vaginally twice daily for endometrial transformation, and oral estradiol was maintained. High-quality SBT was performed on day 5 after endometrial transformation. The luteal support regimen administered after transfer was the same as that administered after endometrial transformation (Fig. 2).

### Thawing and culturing of frozen-thawed embryos

All operations were performed according to the instructions of the vitrification resuscitation kit (Vitrification VT102, Kitazato, Japan). On the morning of the day of transfer, after removing the cannula from liquid nitrogen, the carrier rods were removed and quickly placed in a thawing solution at room temperature for 1 min. The blastocysts were then transferred to dilution solution for 3 min, washing solution 1 for 5 min, or washing solution 2 for 5 min. Finally, the blastocysts were transferred to a blastocyst culture solution for observation and scoring.

### Blastocyst evaluation criteria

Gardner’s grading criteria were used to score all thawed blastocysts [6]. Blastocysts were classified into six stages according to the size of the blastocyst cavity and into three stages (A, B, and C) according to the number and morphology of the inner cell mass and trophoblastic ectodermal cells, respectively. Blastocysts graded as ≥ 3AA, 3AB, 3BA, or 3BB were considered high-quality blastocysts [7].

### Determination of pregnancy outcomes and complications

Pregnancy was determined by β-human chorionic gonadotropin (β-hCG) testing on days 10–12 after FET. The first B-ultrasound was performed 14–16 days after β-hCG testing, and the second B-ultrasound was performed 14–16 days after the first B-ultrasound (Fig. 2). All patients were followed up by regular telephone calls after FET, and outcomes related to the delivery and birth of a newborn were recorded for pregnant women. Biochemical pregnancy was defined as a β-hCG concentration ≥ 15 mlU/mL within one month of FET but no gestational sac on ultrasound. Clinical pregnancy was defined as the presence of a gestational sac in the uterus on ultrasound and a heartbeat. Ectopic pregnancy was defined as the implantation and development of an embryo outside the uterine cavity. Miscarriage was defined as the termination of pregnancy at less than 28 weeks of gestation with a fetus weighing less than 1,000 g [8]. Preterm birth was defined as delivery between 28 and 37 weeks of gestation. Newborns with a birth weight < 2,500 g were considered to have low birth weight, while those with a birth weight ≥ 4,000 g were considered to have macrosomia. Birth defects referred to all kinds of congenital abnormalities in newborns, including abnormalities in body structure, function, metabolism, and development. Gestational hypertension was defined as the first occurrence of hypertension after 20 weeks of pregnancy, a systolic blood pressure ≥ 140 mmHg and/or a diastolic blood pressure ≥ 90 mmHg, and a return to normal within 12 weeks after delivery with a negative urine protein test. Women who had not been diagnosed with diabetes prepregnancy or at their first screening underwent a 75-g oral glucose tolerance test at 24 to 28 weeks of gestation and were diagnosed with gestational diabetes mellitus (GDM) if they met or exceeded any of the following criteria: a fasting blood glucose level of 5.1 mmol/L (92 mg/dL), a 1-h postprandial glucose level of 10.0 mmol/L (180 mg/dL), and a 2-h postprandial glucose level of 8.5 mmol/L (153 mg/dL).

The adverse pregnancy outcomes included in this study were biochemical pregnancy, miscarriage, and ectopic pregnancy. The obstetric and neonatal complications included GDM, gestational hypertension, preterm birth, low birth weight, birth defects, and macrosomia. Non-live birth outcomes included adverse pregnancy outcomes and nonpregnancy.

### Statistical methods

SPSS (version 26.0; IBM, Chicago) statistical software was used for data analysis. Continuous variables were expressed as the mean ± standard deviation, differences among the four groups were compared using one-way analysis of variance (ANOVA), and multiple comparisons between groups were performed using independent t tests. The measured variables were expressed as medians and interquartile ranges (IQRs); the nonparametric Kruskal‒Wallis H test was used to compare differences among the four groups, and the Mann‒Whitney U test was used for multiple comparisons between groups. The chi-square test and Fisher’s exact test were used for categorical variables. Multivariate logistic regression analysis was performed based on the results of the univariate analysis, and the effect of female age on non-live birth outcomes, adverse pregnancy outcomes, or obstetric and neonatal complications were further evaluated after adjusting for mixed factors. Odds ratios (ORs) with 95% confidence intervals (CIs) were calculated for the independent variables, and *P* < 0.05 was considered to indicate statistical significance. Post hoc power analysis was carried out using G-power software (G-power v3.1.9.2, Universitat Kiel, Kiel, Germany).

## Results

### Comparison of baseline characteristics

There were no significant differences in body mass index (BMI), a history of diabetes, hypertension, thyroid disease, male smoking, or endometrial thickness on the day of transformation among the four groups (all *P* > 0.05). Patients in the 38-40-year-old group were the oldest, and those in the < 30-year-old group were the youngest. The differences in maternal age and male partner age among the four groups were statistically significant (all *P* < 0.001). The infertility duration in the < 30-year-old group (3.0 [2.0, 4.0] years) significantly differed from that in the 38-40-year-old group (2.0 [1.0, 4.0] years, *P* = 0.008), 35-37-year-old group (3.0 [2.0, 6.0] years, *P* < 0.001), and 30-34-year-old group (3.0 [1.0, 5.0] years, *P* = 0.002). The proportion of patients with primary infertility in the < 30-year-old group was significantly greater than that in the other three groups (56.5% vs. 8.2%, 18.3%, and 37.5%, respectively; all *P* < 0.001). Among the causes of infertility, the proportion of patients with male factor infertility in the 38-40-year-old group was greater than that in the 30-34-year-old group (21.1% vs. 11.2%, *P* = 0.002) and < 30-year-old group (21.1% vs. 14.0%, *P* = 0.039); the proportion of patients with both female and male factors infertility in the 35-37-year-old group was significantly lower than that in the < 30-year-old group (8.6% vs. 15.4%, *P* = 0.026); and the proportion of patients with unexplained infertility in the < 30-year-old group was the lowest, which significantly differed from that in the 30-34-year-old group (8.7% vs. 13.5%, *P* = 0.017). The proportion of patients who underwent their first transfer cycle in the 35-37-year-old group was the lowest (57.9%), which significantly differed from that in the 30-34-year-old group (69.4%, *P* = 0.007) and the < 30-year-old group (71.1%, *P* = 0.002). The proportion of patients who underwent their first transfer cycle in the < 30-year-old group was the highest (71.1%), which was also significantly different from that in the 38-40-year-old group (61.9%, *P* = 0.034). In terms of the number of births, the proportion of patients with 1–2 births in the 38-40-year-old group was the highest (70.7%), and the proportion of no births in the < 30-year-old group was the highest (87.8%); these differences were statistically significant among the groups. However, there was no significant difference in the proportion of patients with more than 3 births among the four groups. In addition, there were significant differences in the number of pregnancies, miscarriages, and early spontaneous abortions among the four groups (Table 1).

**Table 1:** Comparison of baseline characteristics

	38–40-year-old group (*n* = 147)	35–37-year-old group (*n* = 164)	30-34-year-old group (*n* = 483)	< 30-year-old group (*n* = 485)	*P* value
Maternal age, median(IQR)(year)^U^	38.5(38.0,39.4)^a, b,c^	35.7(35.1,36.4)^d, e^	32.1(30.9,33.3)^f^	27.6(26.0,28.8)	< 0.001^*^
Male age, mean(SD)(year)	39.66 ± 4.61^a, b,c^	37.68 ± 3.62^d, e^	34.05 ± 3.49^f^	29.98 ± 3.41	< 0.001^*^
Infertility duration, median(IQR)(year)^U^	2.0(1.0,4.0)^c^	3.0(2.0,6.0)^d, e^	3.0(1.0,5.0)^f^	3.0(2.0,4.0)	0.060
BMI, median(IQR)(kg/m2)^U^	21.48(20.03,23.31)	21.49(19.98,23.88)	21.10(19.29,23.62)	20.96(19.21,23.44)	0.076
**Infertility type**					
Primary infertility%(n)	8.2(12/147)^a, b,c^	18.3(30/164)^d, e^	37.5(181/483)^f^	56.5(274/485)	<0.001^*^
Secondary infertility%(n)	91.8(135/147)^a, b,c^	81.7(134/164)^d, e^	62.5(302/483)^f^	43.5(211/485)	< 0.001^*^
**Infertile causes**					
Female factor%(n)^#^	57.8(85/147)	65.2(107/164)	62.1(300/483)	61.9(300/485)	0.610
Male factor%(n)	21.1(31/147)^b, c^	15.2(25/164)	11.2(54/483)	14.0(68/485)	0.023^*^
Both factors%(n)^	11.6(17/147)	8.6(14/164)^e^	13.2(64/483)	15.4(75/485)	0.134
Unexplained factor%(n)	9.5(14/147)	11.0(18/164)	13.5(65/483)^f^	8.7(42/485)	0.109
**Transplant cycle**					
First cycle%(n)	61.9(91/147)^c^	57.9(95/164)^d, e^	69.4(335/483)	71.1(345/485)	0.005^*^
Second cycle%(n)	38.1056/147)^c^	42.1(69/164)^d, e^	30.6(148/483)	28.9(140/485)	0.005^*^
**History of diabetes**					
Yes%(n)	0.7(1/147)	0.6(1/164)	0.8(4/483)	0.2(1/485)	0.472
No%(n)	99.3(146/147)	99.4(163/164)	99.2(479/483)	99.8(484/485)	0.472
**History of hypertension**					
Yes%(n)	2.0(3/147)	1.8(3/164)	0.8(4/483)	0.4(2/485)	0.121
No%(n)	98.0(144/147)	98.2(161/164)	99.2(479/483)	99.6(483/485)	0.121
**History of thyroid disease**					
Yes%(n)	0.7(1/147)	0(0/164)	1.5(7/483)	1.9(9/485)	0.324
No%(n)	99.3(146/147)	100(164/164)	98.5(476/483)	98.1(476/485)	0.324
**History of male smoking**					
Yes%(n)	17.0(25/147)	17.1(28/164)	17.0(82/483)	18.6(90/485)	0.919
No%(n)	83.0(122/147)	82.9(136/164)	83.0(401/483)	81.4(395/485)	0.919
**Number of births**					
0%(n)	28.6(42/147)^a, b,c^	49.4(81/164)^d, e^	67.3(325/483)^f^	87.8(426/485)	< 0.001^*^
1–2%(n)	70.7(104/147)^a, b,c^	50.0(82/164)^d, e^	32.1(155/483)^f^	12.2(59/485)	< 0.001^*^
≥ 3%(n)	0.7(1/147)	0.6(1/164)	0.6(3/483)	0(0/485)	0.193
**Number of miscarriages**					
0%(n)	25.2(37/147)^a, b,c^	36.6(60/164)^d, e^	52.4(253/483)^f^	62.1(301/485)	< 0.001^*^
1–2%(n)	51.0(75/147)^b, c^	48.2(79/164)^d, e^	37.5(181/483)	33.2(161/485)	< 0.001^*^
≥ 3%(n)	23.8(35/147)^b, c^	15.2(25/164)^e^	10.1(49/483)^f^	4.7(23/485)	< 0.001^*^
**Number of pregnancies**					
0%(n)	8.2(12/147)^a, b,c^	18.3(30/164)^d, e^	37.5(181/483)^f^	56.5(274/485)	< 0.001^*^
1–2%(n)	51.7(76/147)^c^	54.9(90/164)^d, e^	44.3(214/483)^f^	34.4(167/485)	< 0.001^*^
≥ 3%(n)	40.1(59/147)^a, b,c^	26.8(44/164)^d, e^	16.2(78/483)^f^	9.1(44/485)	< 0.001^*^
**Number of early spontaneous abortion**					
0%(n)	71.4(105/147)^b, c^	73.2(120/164)^e^	80.1(387/483)^f^	85.2(413/485)	< 0.001^*^
1%(n)	19.7(29/147)^c^	18.3(30/164)^e^	14.5(70/483)	12.2(59/485)	0.067
2%(n)	8.9(13/147)^c^	8.5(14/164)^e^	5.4(26/483)^f^	2.6(13/485)	0.003^*^
Endometrial thickness on the transformation day, mean(SD)(mm)	9.06 ± 1.53	9.11 ± 1.48	9.16 ± 1.44	9.27 ± 1.42	0.323

“a” represents *P* value less than 0.05 between 38–40-year-old group and 35–37-year-old group, “b” represents *P* value less than 0.05 between 38–40-year-old group and 30-34-year-old group, “c” represents *P* value less than 0.05 between 38–40-year-old group and <30-year-old group, “d” represents *P* value less than 0.05 between 35–37-year-old group and 30-34-year-old group, “e” represents *P* value less than 0.05 between 35–37-year-old group and <30-year-old group, “f” represents *P* value less than 0.05 between 30-34-year-old group and <30-year-old group

*SD* Standard deviation, *IQR* Inter Quartile Range, *BMI* Body mass index

^#^Female factors mainly include polycystic ovary syndrome, endometriosis, tubal obstruction

^^^Both factors were defined as more than one reason causing infertility

^*^P < 0.05 was statistical significance

^U^:Kruskal-Wallis H test/groups individually tested by Mann-Whitney U-test

### Comparison of pregnancy and neonatal outcomes

There was no significant difference in the biochemical pregnancy rate, miscarriage rate, ectopic pregnancy rate, twin pregnancy rate, preterm birth rate, neonatal birth weight, incidence of macrosomia, incidence of low birth weight, birth defect rate, neonatal sex ratio or obstetric complications among the four groups (all *P* > 0.05). The rates of hCG positivity (59.2%), clinical pregnancy (47.6%), embryo implantation (49.7%) and live birth (34.0%) in the 38-40-year-old group were significantly lower than those in the 30-34-year-old group (74.1%, 64.4%, 65.6%, and 50.9%, respectively; all *P* < 0.001) and the < 30-year-old group (73.2%, *P* = 0.001; 62.9%, *P* = 0.001; 63.9%, *P* = 0.002; 50.7%, *P* < 0.001). The rates of hCG positivity, clinical pregnancy, embryo implantation, and live birth in the 35-37-year-old group were not significantly different from those in the other three groups (all *P* > 0.05). The gestational age at birth in the 35-37-year-old group was the lowest (38.71 ± 1.79 weeks), which significantly differed from that in the 30-34-year-old group (39.23 ± 1.80 weeks, *P* = 0.040) and < 30-year-old group (39.31 ± 1.88 weeks, *P* = 0.021) (Table 2).

**Table 2:** Comparison of pregnancy outcomes and neonatal outcomes

	38–40-year-old group (*n* = 147)	35–37-year-old group (*n* = 164)	30-34-year-old group (*n* = 483)	< 30-year-old group (*n* = 485)	*P* value
Positive rate of hCG test%(n)	59.2(87/147)^b, c^	68.3(112/164)	74.1(358/483)	73.2(355/485)	0.003^*^
Clinical pregnancy rate%(n)	47.6(70/147)^b, c^	56.1(92/164)	64.4(311/483)	62.9(305/485)	0.001^*^
Embryo implantation rate%(n)	49.7(73/147)^b, c^	56.1(92/164)	65.6(317/483)	63.9(310/485)	0.002^*^
Biochemical pregnancy rate%(n)	11.6(17/147)	12.2(20/164)	9.7(47/483)	10.3(50/485)	0.801
Miscarriage rate%(n)	28.6(20/70)	22.8(21/92)	20.9(65/311)	18.4(56/305)	0.273
Ectopic pregnancy rate%(n)	0(0/70)	1.1(1/92)	0(0/311)	1.0(3/305)	0.193
Twin pregnancy rate%(n)	4.3(3/70)	0(0/92)	1.9(6/311)	1.6(5/305)	0.208
Preterm birth rate%(n)	6.0(3/50)	11.4(8/70)	7.7(19/246)	6.9(17/246)	0.672
Live birth rate%(n)	34.0(50/147)^b, c^	42.7(70/164)	50.9(246/483)	50.7(246/485)	0.001^*^
Neonatal birth weight, mean(SD)(g)	3238.65 ± 606.74	3304.26 ± 487.09	3303.35 ± 557.21	3279.90 ± 521.08	0.860
Neonatal birth age, mean(SD)(weeks)	38.95 ± 1.81	38.71 ± 1.79^d, e^	39.23 ± 1.80	39.31 ± 1.88	0.089
Incidence of macrosomia%(n)	1.9(1/52)	7.1(5/70)	9.6(24/251)	6.8(17/251)	0.195
Incidence of low birth weight infants%(n)	7.7(4/52)	2.9(2/70)	6.4(16/251)	8.0(20/251)	0.439
Birth defect rate%(n)	0(0/52)	1.4(1/70)	1.6(4/251)	0.8(2/251)	0.799
**Neonatal sex ratio%(n)**					
Male	67.3(35/52)	51.4(36/70)	58.6(147/251)	53.0(133/251)	0.181
Female	32.7(17/52)	48.6(34/70)	41.4(104/251)	47.0(118/251)	0.181
**Obstetric complications**					
Gestational hypertension%(n)	1.4(1/70)	3.3(3/92)	1.0(3/311)	1.6(5/305)	0.667
GDM%(n)	7.1(5/70)	4.4(4/92)	2.6(8/311)	3.9(12/305)	0.373

*SD* Standard deviation, *GDM* Gestational Diabetes Mellitus

“a” represents *P* value less than 0.05 between 38–40-year-old group and 35–37-year-old group, “b” represents *P* value less than 0.05 between 38–40-year-old group and 30-34-year-old group, “c” represents *P* value less than 0.05 between 38–40-year-old group and <30-year-old group, “d” represents *P* value less than 0.05 between 35–37-year-old group and 30-34-year-old group, “e” represents *P* value less than 0.05 between 35–37-year-old group and <30-year-old group, “f” represents *P* value less than 0.05 between 30-34-year-old group and <30-year-old group

^*^P < 0.05 was statistical significance

### Main factors affecting non-live birth outcomes

The 1,279 females were divided into a live birth group (*n* = 612) and a non-live birth group (*n* = 667) according to whether the live birth outcome was recorded. According to the univariate analysis based on the data of the development cohort, the main factors associated with live birth were infertility duration, BMI, female age, male age, infertility type, the number of transfer cycles, the number of births, the number of miscarriages, and the number of pregnancies.

Multivariate logistic regression analysis led us to exclude BMI, male age, infertility type, the number of births, and the number of pregnancies (*P* values greater than 0.05). Dominant risk factors for non-live birth outcomes included an infertility duration ≥ 3 years (OR = 1.408, 95% CI: 1.049–1.889), 37 < maternal age ≤ 40 years (OR = 2.121, 95% CI: 1.233–3.647), and a history of 1–2 miscarriages (OR = 1.709, 95% CI: 1.057–2.763). A second transfer cycle (OR = 0.772, 95% CI: 0.605–0.986) was found to be a protective factor on live birth outcomes (Table 3).

**Table 3:** Main factors affecting non- live birth outcomes

Factors	Univariable		Multivariable	
	OR (95% CI)	*P* value	OR (95% CI)	*P *value
**Infertility duration (year)**				
< 3	Ref			
≥ 3	1.282(1.029–1.597)	0.027*	1.408(1.049–1.889)	0.023*
**BMI(kg/m** ^**2**^ **)**				
< 18.5	Ref			
18.5 ≤ BMI < 24	1.347(0.968–1.874)	0.077	1.235(0.882–1.730)	0.219
24 ≤ BMI < 28	1.460(0.974–2.187)	0.067	1.361(0.900-2.057)	0.144
≥ 28	1.831(1.007–3.330)	0.047*	1.773(0.967–3.252)	0.064
**Female age(year)**				
< 30	Ref			
30 ≤ Age < 35	0.992(0.771–1.276)	0.948	1.175(0.845–1.633)	0.337
35 ≤ Age ≤ 37	1.382(0.967–1.975)	0.076	1.517(0.944–2.439)	0.085
37< Age ≤ 40	1.997(1.359–2.934)	< 0.001*	2.121(1.233–3.647)	0.007*
**Male age(year)**				
< 30	Ref			
30 ≤ Age < 35	0.941(0.695–1.275)	0.696	0.909(0.649–1.273)	0.578
35 ≤ Age < 40	1.219(0.878–1.693)	0.236	0.930(0.609–1.419)	0.736
≥ 40	1.530(1.012–2.312)	0.044*	0.992(0.572–1.720)	0.977
**Infertility type**				
Primary infertility	Ref			
Secondary infertiliy	1.271(1.015–1.592)	0.037*	0.246(0.024–2.474)	0.234
**Transplant cycle**				
First cycle	Ref			
Second cycle	1.365(1.078–1.729)	0.010*	0.772(0.605–0.986)	0.038*
**PCOS**				
No	Ref			
Yes	1.029(0.783–1.352)	0.839		
**Endometriosis**				
No	Ref			
Yes	0.868(0.459–1.642)	0.663		
**Diabetes**				
No	Ref			
Yes	0.687(0.153–3.081)	0.624		
**Hypertension**				
No	Ref			
Yes	0.917(0.294–2.858)	0.881		
**Thyroid disease**				
No	Ref			
Yes	0.638(0.242–1.688)	0.366		
**Number of births**				
0	Ref			
1–2	1.302(1.026–1.651)	0.030*	1.275(0.857–1.897)	0.232
≥ 3	1.497(0.249–9.001)	0.660	1.419(0.204–9.859)	0.723
**Number of miscarriages**				
0	Ref			
1–2	1.332(1.053–1.683)	0.017*	1.709(1.057–2.763)	0.029*
≥ 3	1.304(0.895–1.899)	0.167	1.530(0.655–3.573)	0.326
**Number of pregnancies**				
0	Ref			
1–2	1.192(0.937–1.517)	0.153	0.158(0.015–1.637)	0.122
≥ 3	1.436(1.040–1.983)	0.028*	0.158(0.013–1.843)	0.141
**Number of early spontaneous abortion**				
0	Ref			
1	1.106(0.810–1.509)	0.527		
2	1.280(0.774–2.117)	0.337		
**Male smoking history**				
No	Ref			
Yes	1.334(0.997–1.786)	0.053		
**Endometrial thickness (mm)**				
< 8	Ref			
8–12	0.815(0.599–1.107)	0.191		
> 12	0.916(0.486–1.726)	0.785		

*BMI* Body Mass Index, *PCOS *Polycystic Ovary Syndrome

*P < 0.05 was statistical significance

### Main factors affecting adverse pregnancy outcomes

Females with β-hCG levels ≥ 15 mlU/mL (*n* = 912) were divided into two groups according to the occurrence of adverse pregnancy outcomes. According to the univariate analysis of the data from the development cohort, the main factors associated with adverse pregnancy outcomes were BMI and female age.

Multivariate logistic regression analysis led us to exclude patients with 18.5 ≤ BMI < 24 kg/m^2^ (*P* values greater than 0.05). The dominant risk factors for adverse pregnancy outcomes were 24 ≤ BMI < 28 kg/m^2^ (OR = 1.856, 95% CI: 1.089–3.163) and 37 < maternal age ≤ 40 years (OR = 1.630, 95% CI: 1.010–2.633) (Table 4).

**Table 4:** Main factors affecting adverse pregnancy outcomes

Factors	Univariable		Multivariable	
	OR (95% CI)	*P* value	OR (95% CI)	*P* value
**Infertility duration (year)**				
< 3	Ref			
≥ 3	1.180(0.895–1.556)	0.241		
**BMI(kg/m** ^**2**3^ **)**				
< 18.5	Ref			
18.5 ≤ BMI < 24	1.621(1.034–2.541)	0.035*	1.544(0.982–2.428)	0.060
24 ≤ BMI < 28	1.925(1.133–3.269)	0.015*	1.856(1.089–3.163)	0.023*
≥ 28	2.026(0.940–4.366)	0.072	2.082(0.965–4.494)	0.062
**Female age(year)**				
< 30	Ref			
30 ≤ Age < 35	1.028(0.748–1.411)	0.867	1.023(0.743–1.407)	0.891
35 ≤ Age ≤ 37	1.361(0.869–2.131)	0.178	1.316(0.837–2.069)	0.235
37< Age ≤ 40	1.649(1.025–2.653)	0.039*	1.630(1.010–2.633)	0.046*
**Male age(year)**				
< 30	Ref			
30 ≤ Age < 35	0.747(0.512–1.090)	0.131		
35 ≤ Age < 40	1.138(0.764–1.694)	0.525		
≥ 40	1.204(0.722–2.009)	0.476		
**Infertility type**				
Primary infertility	Ref			
Secondary infertiliy	1.021(0.771–1.351)	0.886		
**Transplant cycle**				
First cycle	Ref			
Second cycle	1.148(0.851–1.549)	0.367		
**PCOS**				
No	Ref			
Yes	1.200(0.859–1.676)	0.285		
**Endometriosis**				
No	Ref			
Yes	0.502(0.186–1.350)	0.172		
**Diabetes**				
No	Ref			
Yes	1.020(0.186–5.601)	0.982		
**Hypertension**				
No	Ref			
Yes	1.020(0.253–4.108)	0.978		
**Thyroid disease**				
No	Ref			
Yes	1.020(0.346–3.012)	0.971		
**Number of births**				
0	Ref			
1–2	1.050(0.774–1.424)	0.754		
≥ 3	2.076(0.290-14.841)	0.467		
**Number of miscarriages**				
0	Ref			
1–2	1.170(0.872–1.569)	0.296		
≥ 3	0.990(0.604–1.621)	0.967		
**Number of pregnancies**				
0	Ref			
1–2	1.020(0.757–1.376)	0.895		
≥ 3	0.968(0.635–1.475)	0.879		
**Number of early spontaneous abortion**				
0	Ref			
1	1.187(0.807–1.745)	0.383		
2	1.614(0.897–2.902)	0.110		
**Male smoking history**				
No	Ref			
Yes	1.293(0.899–1.858)	0.165		
**Endometrial thickness (mm)**				
< 8	Ref			
8–12	0.890(0.603–1.314)	0.559		
> 12	1.190(0.557–2.543)	0.654		

*BMI* Body Mass Index, *PCOS* Polycystic Ovary Syndrome

*P < 0.05 was statistical significance

### Main factors affecting obstetric and neonatal complications

All patients with live births (*n* = 612) were divided into two groups based on the presence or absence of obstetric and neonatal complications. According to the univariate analysis of the data from the development cohort, the main factors associated with obstetric and neonatal complications were BMI and the number of transfer cycles.

Multivariate logistic regression analysis led us to exclude transplant cycles with *P* values greater than 0.05. The dominant risk factors for obstetric and neonatal complications included 24 ≤ BMI < 28 kg/m^2^ (OR = 2.258, 95% CI: 1.098–4.646) and BMI ≥ 28 kg/m^2^ (OR = 3.431, 95% CI: 1.255–9.381) (Table 5).

**Table 5:** Main factors affecting obstetric and neonatal complications

Factors	Univariable		Multivariable	
	OR (95% CI)	*P* value	OR (95% CI)	*P* value
**Infertility duration (year)**				
< 3	Ref			
≥ 3	1.071(0.708–1.619)	0.746		
**BMI(kg/m** ^**2**^ **)**				
< 18.5	Ref			
18.5 ≤ BMI < 24	1.646(0.890–3.044)	0.112	1.612(0.870–2.986)	0.129
24 ≤ BMI < 28	2.353(1.147–4.826)	0.020*	2.258(1.098–4.646)	0.027*
≥ 28	3.429(1.258–9.345)	0.016*	3.431(1.255–9.381)	0.016*
**Female age(year)**				
< 30	Ref			
30 ≤ Age < 35	1.048(0.686–1.602)	0.829		
35 ≤ Age ≤ 37	1.524(0.842–2.759)	0.164		
37< Age ≤ 40	0.780(0.357–1.706)	0.535		
**Male age(year)**				
< 30	Ref			
30 ≤ Age < 35	1.144(0.688–1.901)	0.605		
35 ≤ Age < 40	0.857(0.479–1.531)	0.601		
≥ 40	1.083(0.518–2.263)	0.832		
**Infertility type**				
Primary infertility	Ref			
Secondary infertiliy	1.146(0.780–1.685)	0.487		
**Transplant cycle**				
First cycle	Ref			
Second cycle	1.526(1.021–2.279)	0.039*	1.495(0.997–2.241)	0.052
**PCOS**				
No	Ref			
Yes	1.327(0.843–2.088)	0.221		
**Endometriosis**				
No	Ref			
Yes	1.858(0.727–4.752)	0.196		
**Diabetes**				
No	Ref			
Yes	3.406(0.475–24.401)	0.223		
**Hypertension**				
No	Ref			
Yes	0.672(0.078-5.800)	0.718		
**Thyroid disease**				
No	Ref			
Yes	2.284(0.635–8.212)	0.206		
**Number of births**				
0	Ref			
1–2	0.958(0.630–1.458)	0.842		
≥ 3	0.918(0.740–1.326)	0.956		
**Number of miscarriages**				
0	Ref			
1–2	1.222(0.815–1.832)	0.331		
≥ 3	1.398(0.743–2.629)	0.298		
**Number of pregnancies**				
0	Ref			
1–2	1.049(0.693–1.587)	0.822		
≥ 3	1.371(0.792–2.372)	0.260		
**Number of early spontaneous abortion**				
0	Ref			
1	1.572(0.947–2.610)	0.080		
2	1.475(0.632–3.444)	0.368		
**Male smoking history**				
No	Ref			
Yes	1.110(0.664–1.855)	0.690		
**Endometrial thickness (mm)**				
< 8	Ref			
8–12	1.182(0.807–1.731)	0.391		
> 12	0.481(0.107–2.159)	0.340		

*BMI* Body Mass Index, *PCOS* Polycystic Ovary Syndrome

*P < 0.05 was statistical significance

Post hoc power calculations indicated that the study sample size yielded > 80% power for the primary outcomes.

## Discussion

In this study, the feasibility of frozen-thawed high-quality SBT in patients of different ages was investigated. The results of the study suggested that women aged 35–37 years who undergo high-quality SBT in an FET cycle can achieve pregnancy outcomes similar to those of younger women. However, for women aged 38–40 years, this transfer strategy did not show the same superiority. To our knowledge, this was the first study to determine the appropriate population for the use of a frozen-thawed high-quality SBT strategy.

It is well known that female age is an independent factor affecting fertility and pregnancy outcomes. In 1958, the International Federation of Gynecology and Obstetrics defined advanced maternal age (AMA) as a maternal age older than 35 years, but it remains to be seen whether the medical community should adopt the same age threshold for women of advanced age. The relevant research evidence in the Chinese Practice Guideline on ART Strategies for Women with Advanced Age defines an age ≥ 35 years as the cutoff for female reproductive age [2]. However, after 35 years of age, the female ovarian reserve is still at a high level until approximately 38 years of age, at which there is a significant decline [9]. Therefore, in the field of ART, some researchers consider female infertility patients aged ≥ 38 years or 40 years as women of advanced age. There are many women of advanced age, individual differences are large, and it is difficult to assist women in achieving pregnancy. Therefore, to improve the clinical outcomes of patients of different ages, it is necessary to formulate individualized transfer strategies.

Age is one of the most important factors in choosing an embryo transfer strategy. A meta-analysis showed that high-quality SET is the first choice for women under 40 years of age [10], which coincides with previous studies by our team [4, 5]. However, some studies suggest that the best pregnancy outcomes can be obtained by transferring a single blastocyst in individuals under 35 years old and 35–37 years old; while, for individuals aged 38–40 years and older, DET has better perinatal outcomes than SET [11]. Some studies have suggested that SBT is a better choice as long as the quality of the blastocyst is high, regardless of age [12]. At this stage, the age cutoff point for SET is controversial, and most studies have demonstrated the superiority of SET by comparing the difference in pregnancy outcomes between patients who undergo SET and DET. In this study, from a different perspective, patients who underwent high-quality SBT in an FET cycle were divided into four groups according to age, and the results showed that there was no significant difference between the 35-37-year-old group and the other three groups in terms of the hCG positivity rate, clinical pregnancy rate, embryo implantation rate or live birth rate. In contrast, there was a significant difference between the 38-40-year-old group, 30-34-year-old group, and < 30-year-old group. High-quality SBT can help women aged 35–37 years achieve the same pregnancy outcomes as younger women, but for women older than 37 years, this strategy may not be as beneficial. A log-binomial regression analysis further showed that an age greater than 37 years was associated with a 2.121-fold increased risk of non-live birth outcomes and that an age of 35–37 years or younger was not associated with non-live birth outcomes, further confirming the feasibility of high-quality SBT for women aged 35–37 years who are undergoing FET cycles. How to select a suitable and efficient transfer strategy for women aged 38–40 years will be our next research direction.

The natural cycle (NC) regimen and the HRT regimen are the two most commonly used endometrial preparation regimens for FET cycles. Most scholars previously believed that there was no significant difference in the live birth rate or clinical pregnancy rate between the NC regimen and the HRT regimen [13, 14]. However, with the rapid development of FET technology, pregnancy outcomes have steadily improved and become satisfactory. Thus, the choice of endometrial protocol has evolved from “achieving a higher pregnancy rate” to “achieving the best pregnancy rate with the safest maternal-fetal outcome.” In recent years, studies have focused on comparing the efficacy and safety of NC and HRT regimens, but unfortunately, large-sample randomized controlled trials comparing different endometrial preparation regimens for FET cycles are lacking. Therefore, there is debate about the optimal endometrial preparation regimen for women undergoing FET cycles. A high-quality study demonstrated that compared with fresh embryo transfer cycles, FET cycles increase the risk of gestational hypertension but did not clarify whether different endometrial preparation protocols are the primary cause [15]. Scholars have further investigated the influence of different endometrial preparation protocols on pregnancy complications and found that the risk of complications such as gestational hypertension and preeclampsia was significantly greater in the HRT group than in the NC group [16, 17]. The reason may be that HRT regimens utilize exogenous estradiol and progesterone for regulation, and ovulation is inhibited, resulting in a lack of corpus luteum [18]. Vasoactive products, such as relaxin and vascular endothelial growth factor, produced by the corpus luteum have protective effects on the maternal cardiovascular system. In addition, some studies have shown that the incidence of adverse pregnancy outcomes, such as ectopic pregnancy and placental abnormalities, are significantly greater in the HRT group than in the NC group [19, 20]. The NC regimen seems to be superior to the HRT regimen in terms of safety, but it is not necessarily the first choice of clinicians in practice. A survey of 64 fertility centers in the United Kingdom showed that 69% of doctors preferred HRT to NC regimens for patients who ovulated regularly [21]. The HRT protocol was chosen for this study because it is more convenient, time-controllable, and flexible for embryo transfer, requires fewer B-ultrasound monitoring times, and has a lower cycle cancellation rate. There was no significant difference in the rates of pregnancy complications, ectopic pregnancy, or abortion among the four groups. Unfortunately, the study was conducted between 2018 and 2021, and the focus of follow-up at that time did not include placental abnormalities. Therefore, it was unclear whether there were differences in the incidence of placental disease among the four groups. It is hoped that future studies can better address the occurrence of placental abnormalities and ensure the safety of mothers and infants as much as possible.

An increase in childbearing age is another factor that affects pregnancy complications and adverse pregnancy outcomes. AMA has been found to be associated with the clustering of metabolic abnormalities during pregnancy, which in turn is associated with an increased risk of adverse pregnancy outcomes [22]. A prospective cohort study based on AMA conducted in 8 public hospitals in China from 2016 to 2021 showed that women of advanced age had a greater risk of adverse pregnancy outcomes than women under 35 years of age, except for postpartum hemorrhage and small for gestational age [23]. Surprisingly, in this study, although the 35-37-year-old group had a lower age at birth than the 30-34-year-old group and the < 30-year-old group (except for the 38-40-year-old group), there were no statistically significant differences in birth weight, the incidence of macrosomia and low birth weight, the rate of prematurity, the rate of birth defects, or the incidence of related obstetric complications among the four groups. Multivariate analysis also confirmed that the 35-37-year-old group was not associated with adverse pregnancy outcomes or obstetric or neonatal complications. This discovery is consistent with the conclusions of many scholars, such as Wang X and Ni Zhixin [24, 25], who further confirmed the feasibility and safety of frozen-thawed high-quality SBT for women aged 35–37 years. Notably, multivariate regression analysis revealed that a maternal age between 38 and 40 years was associated with a 1.63-fold increased risk of adverse pregnancy outcomes but was not associated with obstetric or neonatal complications.

Interestingly, the proportion of male offspring was high in all four groups, especially in the 38-40-year-old group, but there was no significant difference among the four groups. Previous studies have shown that male infants are more likely to be born to women with high-quality blastocysts than low-quality blastocysts [26, 27]. The reason may be that embryos carrying male genetic material generally have more cells and divide faster. Therefore, the reason for the highest proportion of male offspring in the 38-40-year-old group is that women of advanced age have much greater difficulty forming high-quality blastocysts than younger women, which makes male embryos more likely to be selected for high-quality SBT. Newborn sex is a relatively sensitive issue in China, so there is a lack of large sample studies on the sex ratio of ART-conceived infants. Fortunately, the number of babies born after high-quality SBT at this stage accounts for a small proportion of the total population, so it does not currently affect the sex ratio of infants. However, as ART continues to evolve and an increasing number of babies are born through the use of this technology, whether the demographic structure will be affected in the future is a question worthy of attention.

In addition, the risk of no blastocyst formation in women of advanced age due to decreased ovarian function and low oocyte quality was one of the problems we had to face in this study. Therefore, SBT may reduce embryo utilization and overall success rates, increasing time costs and psychological stress. Studies have shown that the optimal number of high-quality embryos from the cleavage stage to blastocyst transfer for women aged 38 years and older exceeds four [28]. In clinical work, psychological concerns about blastocyst culture costs and the risk of no available blastocysts in women of advanced age are often encountered, which may hinder the further promotion of SBT. Based on these factors, combined with the actual situation of patients, it is particularly important to implement effective and flexible individualized transfer strategies.

The findings of this study have a number of practical implications as followed. First, it confirms that the frozen-thawed high-quality SBT is not limited to young patients, but is also applicable to women aged 35–37 years. Second, the embryo transfer strategies for women over 37 years may need to be more diverse and individualized. However, this study also has three major limitations that will hopefully be addressed in future studies. First, the transfer cycle involved was FET, and whether this transfer strategy is suitable for fresh transfer is worth discussing. Second, the study investigated only posttransfer pregnancy outcomes and neonatal outcomes and did not track neonatal intellectual or physical development after birth. Finally, a large multicenter, prospective randomized controlled study is urgently needed to confirm the results of this retrospective single-center study regarding the feasibility of frozen-thawed high-quality SBT in women aged 35–37 years.

## Conclusion

For women aged 35–37 years undergoing FET cycles, high-quality SBT produces the same satisfactory pregnancy outcomes as younger patients and does not increase the incidence of adverse pregnancy outcomes or obstetric and neonatal complications. High-quality SBT may be a feasible and safe transfer strategy for this population, but prospective randomized controlled studies in a larger population are needed to confirm this finding. Unfortunately, this transfer strategy does not allow women aged 38–40 years to achieve the same pregnancy outcomes as younger patients. How to select a suitable and efficient transfer strategy for women aged 38–40 years will be our next research direction. This study has important clinical significance for women aged 35–37 years for choosing safe and effective transfer strategies during FET cycles and could be further promoted.

### Electronic supplementary material

Below is the link to the electronic supplementary material.

Supplementary Material 1

## Data Availability

Data is provided within the supplementary information files.

## References

[1] Racca A, Drakopoulos P, Van Landuyt L (2020). Single and double embryo transfer provide similar live birth rates in frozen cycles. Gynecol Endocrinol.

[2] Jiang L, Chen Y, Wang Q (2019). A Chinese practice guideline of the assisted reproductive technology strategies for women with advanced age. J Evid Based Med.

[3] Glujovsky D, Quinteiro Retamar AM, Alvarez Sedo CR (2022). Cleavage-stage versus blastocyst-stage embryo transfer in assisted reproductive technology. Cochrane Database Syst Rev.

[4] Wu Y, Lu X, Fu Y (2022). Comparison of frozen-thawed embryo transfer strategies for the treatment of infertility in young women: a retrospective study. Peer J.

[5] Wu Y, Lu X, Chen H (2023). Comparison of frozen-thaw blastocyst transfer strategies in women aged 35–40 years: a retrospective study. Front Endocrinol (Lausanne).

[6] Gardner DK, Lane M, Stevens J (2000). Blastocyst score affects implantation and pregnancy outcome: towards a single blastocyst transfer. Fertil Steril.

[7] Oron G, Son WY, Buckett W (2014). The association between embryo quality and perinatal outcome of singletons born after single embryo transfers: a pilot study. Hum Reprod.

[8] Xie X. Beihua Kong,Tao Duan,Obstetrics and Gynecology[M].No.9.Beijing:People’s Medical publishing house,2018:135–137.

[9] Hansen KR, Knowlton NS, Thyer AC (2008). A new model of reproductive aging: the decline in ovarian non-growing follicle number from birth to menopause. Hum Reprod.

[10] Ma S, Peng Y, Hu L et al. Comparisons of benefits and risks of single embryo transfer versus double embryo transfer: a systematic review and meta-analysis. Reprod Biol Endocrinol. 2022;20(1):20. Published 2022 Jan 27. 10.1186/s12958-022-00899-1. 10.1186/s12958-022-00899-1 PMC8793185 35086551

[11] Kissin DM, Kulkarni AD, Kushnir VA, National ART Surveillance System Group (2014). Number of embryos transferred after in vitro fertilization and good perinatal outcome. Obstet Gynecol.

[12] Chen S, Du H, Liu J et al. Live birth rate and neonatal outcomes of different quantities and qualities of frozen transferred blastocyst in patients requiring whole embryo freezing stratified by age. BMC Pregnancy Childbirth. 2020;20(1):655. Published 2020 Oct 29. 10.1186/s12884-020-03353-5. 10.1186/s12884-020-03353-5 PMC7596959 33121448

[13] Ghobara T, Gelbaya TA, Ayeleke RO. Cycle regimens for frozen-thawed embryo transfer. Cochrane Database Syst Rev. 2017;7(7):CD003414. Published 2017 Jul 5. 10.1002/14651858.CD003414.pub3. 10.1002/14651858.CD003414.pub3 PMC6483463 28675921

[14] Mounce G, McVeigh E, Turner K (2015). Randomized, controlled pilot trial of natural versus hormone replacement therapy cycles in frozen embryo replacement in vitro fertilization. Fertil Steril.

[15] Wei D, Liu JY, Sun Y (2019). Frozen versus fresh single blastocyst transfer in ovulatory women: a multicentre, randomised controlled trial. Lancet.

[16] Fu Y, Chen D, Cai B (2022). Comparison of two mainstream endometrial preparation regimens in vitrified-warmed embryo transfers after PGT. Reprod Biomed Online.

[17] von Versen-Höynck F, Schaub AM, Chi YY (2019). Increased preeclampsia risk and reduced aortic compliance with in Vitro fertilization cycles in the absence of a Corpus Luteum. Hypertension.

[18] Singh B, Reschke L, Segars J (2020). Frozen-thawed embryo transfer: the potential importance of the corpus luteum in preventing obstetrical complications. Fertil Steril.

[19] Liu H, Zhang J, Wang B, Kuang Y (2020). Effect of endometrial thickness on ectopic pregnancy in frozen embryo transfer cycles: an analysis including 17,244 pregnancy cycles. Fertil Steril.

[20] Wang F, Wang Q, Song Y (2023). Programmed frozen embryo transfer cycles are associated with a higher risk of abnormal placental development: a retrospective cohort study of singleton live births. Front Endocrinol (Lausanne).

[21] Noble M, Child T (2022). A UK-wide cross-sectional survey of practice exploring current trends in endometrial preparation for frozen-thawed embryo replacement. Hum Fertil (Camb).

[22] Yen IW, Kuo CH, Lin MW, et al. Advanced maternal age-related clustering of metabolic abnormalities is associated with risks of adverse pregnancy outcomes. J Formos Med Assoc Published Online Dec. 2023;14. 10.1016/j.jfma.2023.11.013. 10.1016/j.jfma.2023.11.013 38097427

[23] Zhou Y, Yin S, Sheng Q (2023). Association of maternal age with adverse pregnancy outcomes: a prospective multicenter cohort study in China. J Glob Health.

[24] Wang X, Xiao Y, Tao T (2023). Influence of maternal age on the birthweight of infants delivered from frozen-thawed blastocyst transfer cycles. Front Endocrinol (Lausanne).

[25] Ni ZX, Wan KM, Zhou ZH (2022). Impact of maternal age on Singleton Birthweight in frozen embryo transfer cycles. Front Endocrinol (Lausanne).

[26] Borgstrøm MB, Kesmodel US, Klausen TW (2021). Developmental stage and morphology of the competent blastocyst are associated with sex of the child but not with other obstetric outcomes: a multicenter cohort study. Hum Reprod.

[27] Jia N, Hao H, Zhang C (2022). Blastocyst quality and perinatal outcomes of frozen-thawed single blastocyst transfer cycles. Front Endocrinol (Lausanne).

[28] Xue X, Li W, Li M (2023). Optimal number of high-quality cleavage-stage embryos for extended culture to blastocyst-stage for transfer in women 38 years and older. Gynecol Endocrinol.

